# Lightweight Authentication Protocol for M2M Communications of Resource-Constrained Devices in Industrial Internet of Things

**DOI:** 10.3390/s20020501

**Published:** 2020-01-16

**Authors:** Evangelina Lara, Leocundo Aguilar, Mauricio A. Sanchez, Jesús A. García

**Affiliations:** Facultad de Ciencias Químicas e Ingeniería, Universidad Autónoma de Baja California, Tijuana BC 22390, Mexico; laguilar@uabc.edu.mx (L.A.); mauricio.sanchez@uabc.edu.mx (M.A.S.); garcia.jesus@uabc.edu.mx (J.A.G.)

**Keywords:** Internet of Things, Industrial Internet of Things, authentication, M2M, lightweight, AVISPA, BAN

## Abstract

The Industrial Internet of Things (IIoT) consists of sensors, networks, and services to connect and control production systems. Its benefits include supply chain monitoring and machine failure detection. However, it has many vulnerabilities, such as industrial espionage and sabotage. Furthermore, many IIoT devices are resource-constrained, which impedes the use of traditional security services for them. Authentication allows devices to be confident of each other’s identity, preventing some security attacks. Many authentication protocols have been proposed for IIoT; however, they have high computing requirements not viable to resource-constrained devices, or they have been found insecure. In this paper, an authentication protocol for resource-constrained IIoT devices is proposed. It is based on the lightweight operations xor, addition, and subtraction, and a hash function. Also, only four messages are exchanged between the principals to authenticate. It has a low execution-time and communication-cost. Its security was successfully assessed with the formal methods Automated Validation of Internet Security Protocols and Applications (AVISPA) tool and Burrows–Abadi–Needham (BAN) logic, together with an informal analysis of its resistance to known attacks. Its performance and security were compared with state-of-the-art protocols, resulting in a good performance for resource-constrained IIoT devices, and higher security similar to computational expensive schemes.

## 1. Introduction

Internet of Things (IoT) is a technology where physical objects are empowered with a virtual representation, allowing them to exchange contextual information to coordinate actions, have a prompt and better response to environmental changes, and use their resources efficiently. Additionally, it enables the user to make informed decisions [1,2]. Many applications are looking forward to the benefits of IoT environments, such as those in industry, smart cities, connected medical services, smart farming, smart retail, and smart homes. [3]. IoT devices are composed of sensors and actuators to have awareness and to respond in their environment, and have a method of communication to interact with other devices [4]. These physical objects have a connection to the Internet, directly or through another device, making them available any time and from everywhere in the world to their service clients. However, this connectivity makes them also accessible to adversaries from any place, who can attack a device to access private information such as location, medical, and financial, and use actuators to perform actions that could damage the system or even be a threat to the user’s welfare [5,6,7].

One application domain of IoT is industry. The Industrial Internet of Things (IIoT) consists of sensors, actuators, networks, and services to connect and control production systems. Through the integration in IIoT, supply chains can be monitored and optimized. Machine failures can be detected, avoiding delays in production that cause loss of revenue, and preventing equipment damage and injuries to workers. Additionally, smart products can know their identity, history, documentation, and production process, and can collect information when deployed and used by their customers. IIoT enables flexible, individualized, and resource-saving production. However, because of the ability to monitor and control production systems, IIoT has many security vulnerabilities, including industrial espionage, sabotage, unnoticed use of counterfeit components, propagation of system failures within and across factories, disclosure of private information of customers and employees, physical damage to human operators because of deliberated or unintentional machine failures, etc. [8,9].

Many authentication protocols for IoT have been proposed. Some of them use public-key cryptography, traditional or elliptic curves, such as [10,11,12,13,14]. However, the long keys and complex computations of this type of cryptography make it difficult for its implementation on resource-constrained devices, because of their small memory, and limited power supply [15,16]. There are also less heavyweight proposals [17,18,19,20], which are based on traditional encryption algorithms, such as AES. However, they still have a high impact on the limited memory and processing power of IoT devices. According to [21] measurements, the execution-time of an encryption algorithm is 75.93% greater than a hash function. Whereas the proposed protocol uses only the operations of xor, addition, subtraction, and hash function. Because those functions are lightweight, the scheme does not have a high impact on the limited computing and battery resources of IoT devices.

IIoT integrates the physical world with the digital world. Consequently, attacks in the digital world will have an effect on the physical world and they can affect any part of our daily life. Furthermore, toward ubiquity, many IIoT devices are manufactured as tiny resource-constrained devices, the lifespan of machines on the production floor is of several decades, and it is not always economically possible to replace all legacy machinery with the latest technology [22]. Therefore, security services must also be considered for legacy resource-constrained devices, to prevent adversaries exploit them as the weakest link of the IIoT system.

In this work, the authentication protocol Lightweight Authentication and Key Distribution (LAKD) for machine-to-machine (M2M) communication in IIoT is proposed. It allows a device to be confident of the identity of another device, and distributes a key to be used to achieve other security properties, such as confidentiality and integrity in their exchanged data. LAKD is designed for highly resource-constrained devices, such as those of Class 0 in the classification of resource-constrained devices of the RFC 7228 [23]; according to a survey in 2017, they correspond to the 29.5% of the IoT devices on the market [24]. To be suitable to them, the proposal is based only on the lightweight operations of xor, addition, and subtraction, and a one-way hash function; thus, the protocol does not have a high impact on the device’s computational and battery resources. It only requires four messages to be exchanged between the principals for the mutual authentication and key distribution. The security of the proposal was assessed through formal and informal methods. It was formally evaluated with the broadly-accepted instruments AVISPA tool [25,26] and Burrows–Abadi–Needham (BAN) logic [27], which showed that it achieves mutual authentication, and it is resistant to the attacks of replay and man-in-the-middle (MITM). Additionally, an informal analysis was done showing the proposed attack-resistance to known attacks, such as tracking, off-line identity guessing, impersonation, injection, MITM, privileged insider, replay, known session-specific temporary information, denial-of-service (DoS), de-synchronization, and key disclosure. The computing requirements and security of LAKD were compared with lightweight schemes, resulting in the proposal having a good execution-time and communication-cost for IIoT, and higher security. The protocol was inspired by the work in [22], wherein an authentication protocol for M2M communication in IIoT was proposed. That only uses the operations xor and hash function; thus, it is very lightweight. However, many vulnerabilities have been found in the protocol [28,29,30].

The rest of the paper follows this organization: Section 2 and Section 3 contain related work and preliminaries, respectively. In Section 4 the proposed LAKD protocol is described. Security analysis is presented in Section 5. Performance and security evaluation is done in Section 6. Section 7 presents the discussion. Finally, Section 8 contains the conclusions.

## 2. Related Work

In this section, current proposals of authentication protocols for IIoT and IoT are briefly analysed.

An authentication protocol for ad hoc wireless sensor networks (WSN) is proposed in [31]; it uses hash functions and xor operations, and considers a heterogeneous environment with two or more types of nodes: tiny resource-constrained sensor nodes, and more powerful gateways. The scheme is examined in [32] and it is found vulnerable to off-line identity guessing, off-line password guessing, stolen smart card, and impersonation attacks. Then, a modified version was proposed, which was analyzed in [33], revealing it does not achieve mutual authentication and does not resist session key disclosure, tracking, and node and user forgery attacks. In [34] the protocol in [31] is also inspected, showing it does not provide user anonymity, and if a node is compromised, it allows the adversary to obtain the session key between the user and another node. Then, they proposed an authentication protocol for ad hoc WSN. The proposal is lightweight because it only relies on xor and hash functions. However, in [35] the authors examine the scheme and show that it is vulnerable to unknown key-share attack, node capture attack, and node information secrecy. Afterward, they propose an authentication protocol for IIoT, which is also lightweight. The scheme does not use any timestamp because of the challenge of synchronizing time in distributed networks; however, in [28] impersonation and DoS attacks are achieved because of the independence of time in an authentication protocol.

Two protocols for ad hoc WSN are proposed in [36]. One of them is lightweight, and the other is more complex because it uses public-key cryptography and it was proposed for hostile environments with a high probability of node capture attacks. In [37], an analysis of both schemes is presented, demonstrating that they do not achieve a proper mutual authentication; the identity of the user is not verified; thus, the protocol is executed even if he/she inputs a wrong identity, and they are vulnerable to stolen smart card and tracking attacks. Then, they propose an authentication scheme for IIoT to resolve the security weakness, achieve resistance to mobile device loss attack, and have user anonymity. The protocol uses encryption, and public-key cryptography on the user’s mobile device; thus, it is not proper for resource-constrained systems [15,16].

In [38], an authentication, authorization and accounting (AAA) system for IIoT is proposed. It is based in the Next Generation Access Control (NGAC) standard [39], a flexible infrastructure to provide access control in different environments. The system provides access control to IIoT devices in a local cloud with security and interoperability, and allows the integration of the inherently heterogeneous IoT devices. Their authentication mechanism uses X.509 certificates, which makes the solution prohibitive for resource-constrained devices, because the complex validation of the public keys exhaust the limited resources of the devices, and the public-key certificate management causes performance bottlenecks [40].

The previous schemes were examined when developing this work to try to achieve a protocol that does not have the aforementioned security issues, and with computing requirements that are not prohibitive to resource-constrained devices. The proposed protocol uses only simple operations to be lightweight. And for security, every transmitted message uses confidential and integrity mechanisms, such as xor-ing and hashing, keeping the secrecy of the transmitted data and resisting malicious modifications.

## 3. Preliminaries

In this section, the network architecture considered in the proposal is described, which follows a vision of energy efficiency in the sensor nodes. Also, the threat model in which the protocol must be secure is presented.

### 3.1. Network Architecture

**Figure 1:**
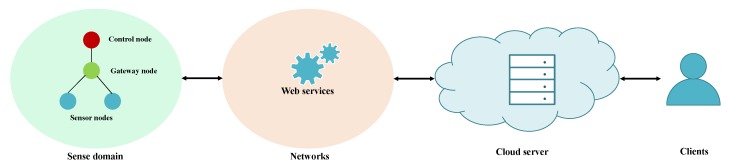
Energy-efficient architecture for the Industrial Internet of Things (IIoT).

The network architecture follows the perspective of IIoT presented in [41,42]. The architecture is illustrated in Figure 1, and it consists of a sensing domain, RESTful services, cloud server, and user applications. The sense domain comprises three layers: sense, gateway, and control. The sensor nodes in the sensing layer are resource-constrained devices responsible for collecting and sending contextual data to its gateway. The gateways store the received data in buffers and forward them to control nodes, which aggregate the information of different gateways and redirect it to networks with RESTful services. These networks act as a bridge between the physical objects in the sense domain and their virtual representation in the cloud. Through organizing the sense domain in the hierarchical framework of three layers, the traffic load and energy consumed by the massive number of sensor nodes can be balanced, increasing the lifetime of the whole IIoT system. The perspective of the architecture is energy efficiency, to achieve a *green* IIoT [41].

This work centers on the resource-constrained devices of the sensing layer, which are unable to use traditional security services to protect their communication with the gateway. Due to the sensing domain consisting of a massive number of sensing nodes, the IIoT system may consume a considerable amount of energy. Through optimizing the sensing, processing, and communicating tasks of the sensing devices, the consumed energy can be reduced [41]. The proposed protocol follows the perspective of low computing-resource usage (low processing-time and communication-cost) on the sensor devices, decreasing the energy cost of the IIoT system and prolonging the battery life of the sensor devices.

### 3.2. Threat Model

The following assumptions about security properties and adversary abilities are made. The notation A represents the adversary.

1. The private channel used for registration is secure.
2. The one-way hash function is collision-resistant.
3. The gateway and sensor node have protection against tampering.
4. A cannot guess random numbers and keys in polynomial time.
5. Replay attack: A can capture messages from old authentication sessions and replay them in the current session.
6. Modification attack: A can tamper intercepted messages.
7. Tracking attack: A can trace the sensor node behind authentication sessions.
8. Impersonation attack: A can pretend to be a legitimate gateway or sensor node.
9. Injection attack: A can send counterfeit messages.

## 4. LAKD Protocol

The proposed protocol consists of two phases: (1) registration, where sensor node and gateway exchange secrets that they will later use to prove their identities, and (2) mutual authentication, where they authenticate and generate a session key. The notation used in the description of the protocol are presented in Table 1.

### 4.1. Registration

In this procedure, the sensor node and gateway exchange secrets that they will later use in the authentication process to prove their identities. First, a sensor node chooses its first pseudonym AID; then, using a secure channel, the sensor node and gateway do the following:

1. The sensor node selects a random number $r_{0}$ and computes: $b0=h(ID_{s}||k_{s}||r_{0})$. Then, it sends to the gateway: AID, $r_{0}$, and b0.
2. Gateway selects a random number $r_{1}$ and computes: $b1=h(ID_{g}||k_{g}||r_{1})$. It selects a key pool KP, whose size will depend on the resource capacity of the sensor node; a KP of three keys could be suitable for resource-constrained devices. Then to the sensor node it sends: b1 and KP.
3. The sensor node and gateway store the values $\left\{b0,b1,KP\right\}$ in a secure manner.

The reason why the sensor node sends $r_{0}$ to the gateway is that there could exist an authentication server who knows all the sensor nodes that are valid in the system. Then, the gateway sends $r_{0}$ and b0 to the authentication server, which computes a $b0^{\prime }$ for each of the valid sensor nodes, using their $ID_{s}$ and $k_{s}$ that it knows, and the received $r_{0}$, until a match between b0 and $b0^{\prime }$ is found. If it does not occur, the authentication server will indicate to the gateway not to register that sensor node in its network because it is not valid.

### 4.2. Authentication

**Figure 2:**
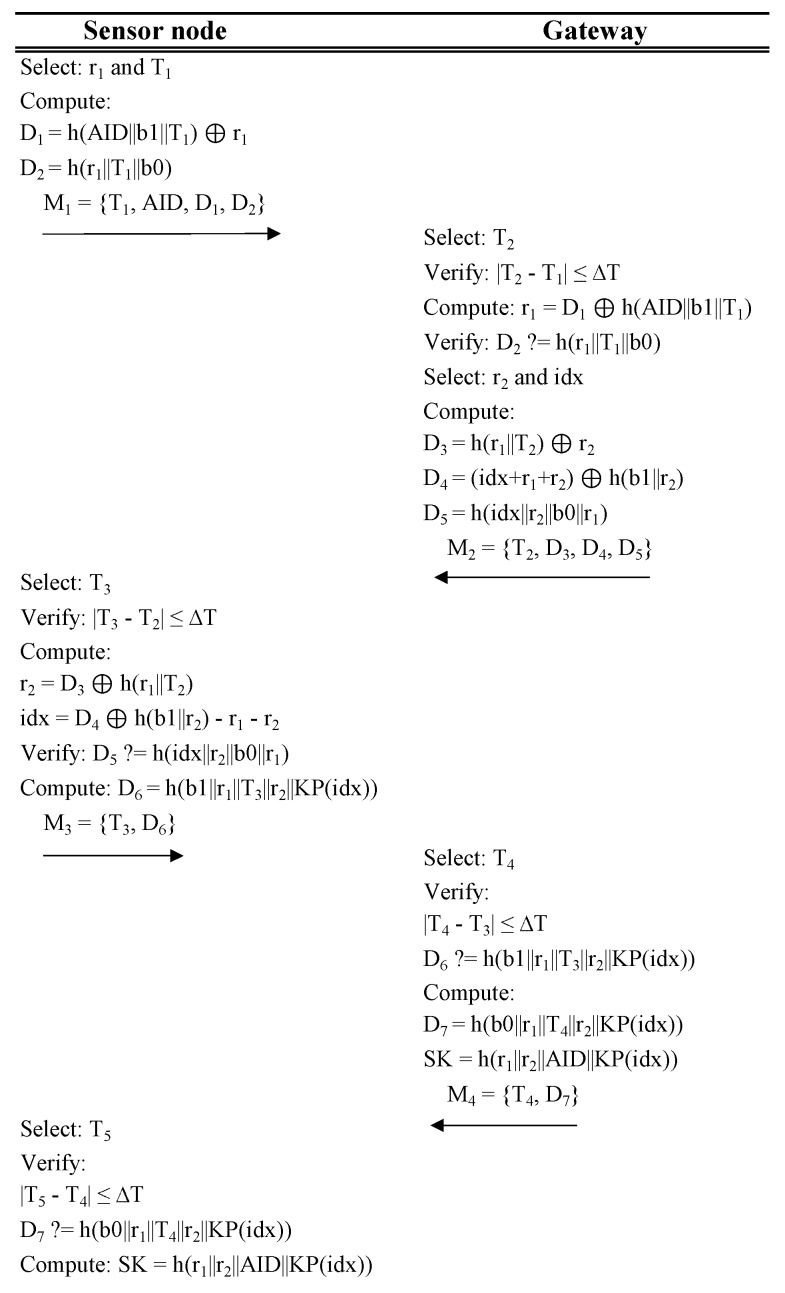
Authentication procedure of LAKD protocol.

The authentication procedure allows the sensor node and gateway to identify each other and have the confidence that they believe in each others’ identities. It consists of five steps, as is described in Figure 2.

1. The sensor node selects a random number $r_{1}$, and a timestamp $T_{1}$, and computes the following: $D_{1}=h(AID||b1||T_{1})⊕r_{1}$ and $D_{2}=h(r_{1}\left|\right|T_{1}\left||b0\right)$. Then, it sends to the gateway $M_{1}=\left\{T_{1},AID,D_{1},D_{2}\right\}$.
2. The gateway selects a timestamp $T_{2}$ and verifies $|T_{2}-T_{1}|\leq \Delta T$; if true it uses its set of values $\left\{b0,b1,KP\right\}$ associated to AID to compute $r_{1}=D_{1}⊕h(AID||b1||T_{1})$ and verify $D_{2}\overset{?}{=}h(r_{1}\left|\right|T_{1}\left||b0\right)$. If any of the verifications are false, the gateway aborts the communication. If true, it selects a random number $r_{2}$, and idx, which is a valid index in the key pool KP of the sensor node, and computes: $D_{3}=h(r_{1}\left|\right|T_{2})⊕r_{2}$, $D_{4}=\left(idx+r_{1}+r_{2}\right)⊕h(b1||r_{2})$, and $D_{5}=h(idx||r_{2}\left||b0|\right|r_{1})$. Then, it sends to the sensor node $M_{2}=\left\{T_{2},D_{3},D_{4},D_{5}\right\}$.
3. The sensor node selects a timestamp $T_{3}$ and verifies $|T_{3}-T_{2}|\leq \Delta T$, if true it computes $r_{2}=D_{3}⊕h(r_{1}\left|\right|T_{2})$, and $idx=D_{4}⊕h(b1||r_{2})-r_{1}-r_{2}$, and verifies $D_{5}\overset{?}{=}h(idx||r_{2}\left||b0|\right|r_{1})$. If any of the verifications are false, sensor node aborts the communication. If true it computes $D_{6}=h(b1||r_{1}\left|\right|T_{3}\left|\right|r_{2}\left||KP\left(idx\right)\right)$, where KP(idx) is the key at index idx of the sensor node key pool KP. Then, it sends to the gateway $M_{3}=\left\{T_{3},D_{6}\right\}$.
4. The gateway selects a timestamp $T_{4}$ and verifies $|T_{4}-T_{3}|\leq \Delta T$; if true it verifies $D_{6}\overset{?}{=}h(b1||r_{1}\left|\right|T_{3}\left|\right|r_{2}\left||KP\left(idx\right)\right)$ using the key in KP at the index it selected in Step 2. If any of the verifications are false, the gateway aborts the communication. If true, it computes $D_{7}=h(b0||r_{1}\left|\right|T_{4}\left|\right|r_{2}\left||KP\left(idx\right)\right)$, and the session key $SK=h(r_{1}||r_{2}||AID||KP\left(idx\right))$. Then, it sends to the sensor node $M_{4}=\left\{T_{4},D_{7}\right\}$.
5. The sensor node selects a timestamp $T_{5}$ and verifies $|T_{5}-T_{4}|\leq \Delta T$; if true it verifies $D_{7}\overset{?}{=}h(b0||r_{1}\left|\right|T_{4}\left|\right|r_{2}\left||KP\left(idx\right)\right)$. If any of the verifications are false, the sensor node does not perform any more computations. If true, it computes $SK=h(r_{1}||r_{2}||AID||KP\left(idx\right))$. Then, the sensor node pseudonym is updated in this manner: $AID=h(AID||b0||r_{1}||r_{2})$.

## 5. Security Analysis

In this section, the security of the proposed protocol is analysed through formal and informal methods. The formal methods consist of AVISPA tool and BAN logic. Both are broadly-accepted instruments to analyse the accomplishment of mutual authentication and resistance to known attacks, and they have been used to assess the security of many protocols, including [43,44,45]. The informal method consists of analysing the security properties achieved by the proposal, and its resistance against known attacks, as was also done in the above-cited works.

### 5.1. Formal Verification with AVISPA

A formal security verification was performed on LAKD protocol using the SPAN+AVISPA tool. The AVISPA tool is an automated verification tool for cryptographic protocols. It supports four back-ends that search for attacks on the security properties of the protocol under verification. They are On-the-fly Model-Checker (OFMC), Constraint-Logic-based Attack Searcher (CL-AtSe), SAT-based Model-Checker (SATMC), and Tree Automata based on Automatic Approximations for the Analysis of Security Protocols (TA4SP) [46]. For the verification, the protocol has to be described using the High-Level Protocol Specification Language (HLPSL). It is a role-based language, where each role describes the information a principal can use initially, such as preshared keys and cryptographic algorithms, the initial state, requirements for state transitions, and specifications concerning how the roles interact with one another [47]. The SPAN tool is a protocol animator for HLPSL specifications which allows interactively building message sequence charts of the protocol and attacks that are found [48].

The AVISPA tool implements a communication channel controlled by a Dolev–Yao intruder, which means that the adversary can intercept, decompose, reassemble or modify the transmitted messages; however, because perfect cryptography is assumed, the adversary can analyze intercepted messages only if he/she possesses the decryption keys [49,50]. Three verifications are performed by the AVISPA tool. The first verification is the executability of a non-trivial HLPSL specification, which ensures that the protocol executes to completion; thus, it can reach a state where possible attacks can be found. The second is the verification of replay attacks, where the back-ends give the intruder the knowledge of regular sessions between legitimate agents, verify if legitimate participants can execute the protocol by searching for a passive intruder, and determine whether a replay attack exists. The third verification is Dolev–Yao checking, where back-ends verify if a MITM attack is possible. After the verifications, the AVISPA tool outputs whether the protocol is concluded safely or unsafely against MITM and replay attacks, or if the analysis is inconclusive [51].

The security goals specified in the HLPSL modeling of LAKD protocol were mutual authentication and secrecy of the session key SK. The first involves that an agent is correct in believing the aimed principal is in the current session, has reached a particular state, and agrees on some value that cannot be used twice with the same participants. If the mutual authentication goal is violated or the intruder learns a secret value, the tool concludes the protocol as unsafe, indicates which goal was unsatisfied, and provides an attack trace which shows the sequence of messages resulting in an attack.

**Figure 3:**
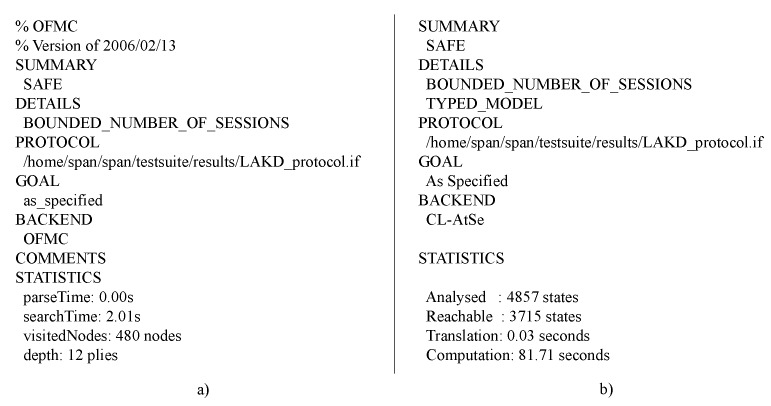
AVISPA verification results: (**a**) Using OFMC back-end. (**b**) Using CL-AtSe back-end.

In the verification of LAKD, CL-AtSe and OFMC back-ends were used because of their support of the xor operation [52]. In Figure 3 the verification results are shown; as can be seen, both back-ends concluded that LAKD protocol is safe against replay and MITM attacks, and the secrecy and mutual authentication goals were achieved.

### 5.2. Formal Verification with BAN Logic

BAN logic is a formal logic for analyzing the security properties of a protocol. It consists of a set of rules and postulates to reason about what principals believe about whom. BAN logic allows verifying whether the exchanged information on an authentication protocol is trustworthy [27]. Its notations and inference rules are introduced in Table 2 and Table 3, respectively.

The security goals LAKD protocol has to satisfy to achieve mutual authentication are the following. GW represents the gateway, and SN the sensor node.

Goal 1: $GW|\equiv (GW\overset{SK}{⟷}SN)$.
Goal 2: $SN|\equiv (GW\overset{SK}{⟷}SN)$.
Goal 3: $GW|\equiv SN|\equiv (GW\overset{SK}{⟷}SN)$.
Goal 4: $SN|\equiv GW|\equiv (GW\overset{SK}{⟷}SN)$.

The idealized form of LAKD protocol is as follows:

Message 1: ${\left\{r_{1},T_{1}\right\}}_{b1},{\left\{{\langle GW\overset{r_{1}}{⇌}SN\rangle }_{b0},T1\right\}}_{b0}$.
Message 2: ${\left\{r_{2},T_{2}\right\}}_{r_{1}},{\left\{{\langle GW\overset{r_{2}}{⇌}SN\rangle }_{r_{1}}\right\}}_{b1},{\left\{{\langle GW\overset{KP(idx)}{⇌}SN\rangle }_{r_{1}},r_{2}\right\}}_{b0}$.
Message 3: ${\left\{GW\overset{SK}{⟷}SN,T_{3}\right\}}_{b1}$.
Message 4: ${\left\{GW\overset{SK}{⟷}SN,T_{4}\right\}}_{b0}$.

The assumptions about the LAKD protocol initial state are the following:

**Assumption** **1.** $GW|\equiv SN\Rightarrow (r_{1},T_{1},T_{3})$ *.*

**Assumption** **2.** $GW|\equiv ♯(T_{1},T_{3})$ *.*

**Assumption** **3.** $GW|\equiv GW\overset{KP(idx)}{⟷}SN$ *.*

**Assumption** **4.** $GW|\equiv GW\overset{b0}{⟷}SN$ *.*

**Assumption** **5.** $GW|\equiv GW\overset{b1}{⟷}SN$ *.*

**Assumption** **6.** $SN|\equiv GW\Rightarrow (r_{2},T_{2},T_{4})$ *.*

**Assumption** **7.** $SN|\equiv ♯(T_{2},T_{4})$ *.*

**Assumption** **8.** $SN|\equiv GW\overset{KP(idx)}{⟷}SN$ *.*

**Assumption** **9.** $SN|\equiv GW\overset{b0}{⟷}SN$ *.*

**Assumption** **10.** $SN|\equiv GW\overset{b1}{⟷}SN$ *.*

The BAN logic proof demonstrating that LAKD protocol achieves the mutual authentication goals is presented in Appendix A. It consists of applying the BAN logic rules to the idealized form of the protocol and initial assumptions.

### 5.3. Informal Security Analysis

In this section, we provide an analysis of the security properties achieved by LAKD protocol and its resistance against known attacks.

#### 5.3.1. Confidentiality

All information which has to be kept confidential between a gateway and sensor node is sent ciphered through an xor, or masked using random numbers and a one-way hash function. To send $r_{1}$ with confidentiality, it is ciphered through an xor with $h(AID||b1||T_{1})$. The value b1 is a secret between sensor node and gateway; therefore, the adversary cannot compute $h(AID||b1||T_{1})$ to obtain $r_{1}$; additionally, it cannot invert the hash function to obtain b1. The use of $T_{1}$ and AID causes the hash value to be different in each protocol execution, preventing the adversary to reuse old values of $h(AID||b1||T_{1})$ seized by him/her. In a similar manner, $r_{2}$ is sent ciphered with $h(r_{1}||T_{2})$, and idx with $h(b1||r_{2})$. The values $r_{1}$ and $r_{2}$ become session secrets. They are used to mask long-term secrets, such as b0, b1, and KP(idx), make messages dependent to the current session, make messages different and unpredictable between sessions, and construct the session key SK. Together with the hash function, they prevent the adversary from discerning secrets from the transmitted messages.

#### 5.3.2. Data Integrity

The following scenarios describe what would happen if a message were to be modified during transmission. In all of them, the receptor can detect a data integrity violation. Consequently, it aborts the communication.

- If $T_{1}$, AID, or $D_{1}$ is modified, the verification of digest value $D_{2}=h(r_{1}\left|\right|T_{1}\left||b0\right)$ will not be true due to it is created with $T_{1}$, $r_{1}$, which the gateway derive from $D_{1}$, and b0 which is selected according to the value of AID. Even if only one of $T_{1}$, AID, or $D_{1}$ is altered, it will cause $r_{1}$ to be different from that originally sent, resulting in $D_{2}$ verification being false. Also, it is infeasible the adversary be able to fabricate a valid $D_{2}$ to hide its modifications, because its construction uses b0, which is a secret between sensor node AID and the gateway.
- If $T_{2}$, $D_{3}$, or $D_{4}$ is modified, the verification of digest value $D_{5}=h(idx||r_{2}\left||b0|\right|r_{1})$ will not be true because it is created with $r_{2}$ and idx, which the gateway tries to derive from $D_{3}$ and $D_{4}$, respectively. Concerning $T_{2}$, it is used in $D_{3}$; thus, its alteration also affects $r_{2}$. Even if only one of $T_{2}$, $D_{3}$, or $D_{4}$ is modified, it will result in different values of $r_{2}$ and idx from the sent by the gateway, causing the verification of $D_{5}$ to be false. Also, it is infeasible the adversary be able to fabricate a valid $D_{5}$, because it uses b0.
- If $T_{3}$ or $D_{6}$ is modified, the verification of digest value $D_{6}=h(b1||r_{1}\left|\right|T_{3}\left|\right|r_{2}\left||KP\left(idx\right)\right)$ will be false, because it is constructed with $T_{3}$. It also uses KP(idx), which is the key from KP selected by gateway through idx in $D_{4}$. It is infeasible the adversary be able to fabricate a valid $D_{6}$, because he/she does not know the secret keys.
- If $T_{4}$ or $D_{7}$ is modified, a similar situation to that previously described will happen, due to $D_{7}$ depends on $T_{4}$ and KP(idx).

#### 5.3.3. Mutual Authentication

In the registration phase of LAKD protocol, the gateway and sensor node construct and exchange in a secure manner, the values $b0=h(ID_{s}||k_{s}||r_{0})$, $b1=h(ID_{g}||k_{g}||r_{1})$, and KP. The knowledge of b0, b1, and KP demonstrates to the gateway that an agent is the sensor node, and vice versa. Gateway authenticates the sensor node by verifying that $M_{1}$ was constructed using b0 and b1, and $M_{3}$ with b1 and KP(idx). The sensor node authenticates the gateway by confirming $M_{2}$ was created with b0 and b1, and $M_{4}$ with b0 and KP(idx). Therefore, both principals authenticate each other.

#### 5.3.4. Sensor Node Anonymity

After each authentication session, the sensor node pseudonym AID is updated, and because random numbers are used in its modification, its new value is unpredictable. Therefore, an adversary can neither know the specific node behind some action nor keep a record of the activities performed by the same node.

#### 5.3.5. Perfect Forward and Backward Secrecy

If the adversary obtains the current session key SK, he/she cannot derive older or futures SKs from it, because there is no relationship between them, since each key is constructed with values specific to the session it belongs. SK is constructed using ephemeral random values sent during the current session, the current AID of the sensor node, and the key KP(idx) selected in the current session. Even if the adversary obtains long-term keys, he/she will not be able to generate older SKs because of the ephemeral random numbers. Therefore, a sensor node can only access information which was transmitted when it was part of the network, neither the previous nor the future one.

#### 5.3.6. Known Session Key Security

Each execution of a key agreement protocol should provide a unique session key. As a result, if the adversary learns some, he/she is not able to generate others [53]. In the proposed protocol, SK is constructed using two random numbers ($r_{1}$ and $r_{2}$), which are ephemeral and different per session. The AID of the sensor node is also used, which changes after each authentication session, and for its modification uses the secret b0 unknown to the adversary. Therefore, the adversary is not able to create SKs even if he/she learned some old ones due to the unpredictable changes.

#### 5.3.7. Resistance to the Tracking Attack

In this attack, an adversary intercepts messages of different sessions and tries to find a relationship between them to determine if they belong to the same sensor node [54,55]. In LAKD protocol, the principals never use their identities. To authenticate, they use the secrets b0 and b1, which are the hash outputs of their identities, keys, and random numbers. Thus, the adversary cannot invert them to know the specific node behind a message. Also, every message is constructed using random numbers. Because they are different per session, messages between sessions are different and unpredictable. Finally, in each session, a different KP(idx) is selected, which contributes to the unpredictability among sessions. Consequently, an adversary cannot track a node from captured messages.

#### 5.3.8. Resistance to the Offline Identity Guessing Attack

In this attack, the adversary tries to guess the sensor node or the gateway identity off-line. In the proposed protocol, only the values b0 and b1 contain the identities of the sensor node and the gateway, respectively. The values are not invertible because they are the result of a hash function. Also, they are never sent in cleartext, when used they are masked with timestamps and unknown random numbers to the adversary, and then input to a hash function. As a result, it is infeasible to obtain the identities or even b0 or b1 from the messages.

#### 5.3.9. Resistance to Impersonation Attack

Every message sent in LAKD protocol uses at least one secret: b0, b1, or KP(idx). They belong to sensor node AID. It is not feasible that an adversary fabricates valid messages $\left(M_{1},M_{3}\right)$ to impersonate sensor node AID, or $\left(M_{2},M_{4}\right)$ to impersonate the gateway, due to the secrecy of b0, b1, and KP(idx), and because of the value AID changes unpredictably in each authentication session. Additionally, in every protocol execution, a different KP(idx) is selected from the key pool. This makes the impersonation even less possible, because the adversary is also required to know all the keys of KP.

#### 5.3.10. Resistance to the Injection Attack

In this attack, an adversary fabricates and sends counterfeit messages to legitimate nodes. The proposal resists this attack like how it does to impersonation attack. Every transmitted message uses at least one shared secret between sensor node and gateway. It is infeasible than an adversary fabricates valid messages without knowing them. Additionally, every message contains a digest of the data in it. Therefore, the sensor node and gateway can easily detect counterfeit, replayed, or modified messages. The digest consists of the timestamp, random numbers, and shared secrets input to a hash function. The adversary cannot invert the function to fabricate a valid digest, also cannot modify the hash output in a deterministic manner to make it valid to his/her counterfeit data.

#### 5.3.11. Resistance to the MITM Attack

In this attack, an adversary deceives agents into thinking their communication is secure, while he/she is in the middle of maliciously modifying and relaying messages. In the LAKD protocol, it is not feasible that the adversary modifies or fabricates messages without the sensor node and gateway detecting it, as described in subsections Data Integrity and Resistance to the Injection Attack. Also, it is not feasible he/she obtains secret values, such as identities, keys, b0, b1, or KP(idx) from the messages sent, because they are masked with random numbers, timestamps, and hash functions. Finally, the adversary is not able to obtain SK, because it is constructed using secret random numbers and the selected KP(idx) in the current protocol execution.

#### 5.3.12. Resistance to the Privileged Insider Attack

In this attack, a privileged insider (e.g., a system manager with access to the gateway) tries to impersonate a node when accessing other servers where that node is registered, using the node’s credentials of its system [56]. In the proposal, the gateway does not know the $ID_{s}$ and $k_{s}$ of the sensor node, and the sensor node does not know the $ID_{g}$ and $k_{g}$ of the gateway. During the registration phase, the principals share those values masked with random numbers and hash functions. Therefore, none of them can invert the operations and obtain the original values to use them to impersonate the node in other systems.

#### 5.3.13. Resistance to the Replay Attack

To perform this attack, the adversary intercepts valid messages and maliciously delays or repeats them, faking ownership over them. LAKD protocol resists this attack in two ways. The first one is using timestamps for the verification of the transmission delay. Messages from an old session will have a transmission delay longer than the allowed. This will be detected by the gateway in $D_{2}$, and by the sensor node in $D_{5}$. Therefore, the receptor will not accept the messages, and it will abort the communication. The second form of resistance against this attack is using the ephemeral random numbers $r_{1}$ and $r_{2}$, and a different KP(idx) key per session. If the adversary replays old messages from the gateway, sensor node will detect in $D_{5}=h(idx||r_{2}\left||b0|\right|r_{1})$ that $r_{1}$ is not the one it sent in $M_{1}$. Similarly, if the replayed messages are from the sensor node, the gateway will detect in $D_{6}=h(b1||r_{1}\left|\right|T_{3}\left|\right|r_{2}\left||KP\left(idx\right)\right)$ that $r_{2}$ is not the one it sent in $M_{2}$, and KP(idx) is not the one it selected. Therefore, LAKD protocol is resistant to replay attacks.

#### 5.3.14. Resistance to the Known Session-Specific Temporary Information Attack

In this attack, the session key secrecy is compromised of the exposition of session-temporal secrets, such as random numbers. The adversary can perform the attack because random numbers are not usually stored in protected memory, as is done with keys and other long-term secrets. If after the session execution, the random numbers are not correctly deleted from memory, or the adversary controls the random number generator, he/she can obtain them and use them to generate the session key [53,57]. LAKD protocol is resistant to this attack, due to SK being constructed using the secret key KP(idx). Even if the adversary obtains the random numbers $r_{1}$ and $r_{2}$, he/she will not be able to generate SK because he/she does not have access to the key pool.

#### 5.3.15. Resistance to the DoS Attack

LAKD protocol can detect intent to perform a DoS attack from the first transmitted message. If $M_{1}$ is a replay message, the gateway will detect that the cleartext $T_{1}$ is not within the allowed transmission-delay. If $T_{1}$ was modified to make it look as valid, then the verification of the digest $D_{2}=h(r_{1}\left|\right|T_{1}\left||b0\right)$ will be false. The adversary cannot construct a valid $D_{2}$ because he/she does not know b0. If $M_{1}$ is not a replay but a fresh and valid message originated by the adversary, the gateway will detect the malicious behavior when receiving many messages with the same AID. If the adversary modifies the cleartext AID to pretend that the message is from a different source, then the verification of $D_{2}$ will be false, because the adversary does not know the b0 of that AID node.

#### 5.3.16. Resistance to the Desynchronization Attack

In this attack, the adversary tries to impede the communication between two legitimate nodes through de-synchronize them in the values required for the authentication [58]. In LAKD protocol, the only value that is updated after an authentication session is the pseudonym AID. In its modification $r_{1}$, $r_{2}$, b0, and the current AID are used. Before updating, the principals verify the identities of each other, and the correctness of the random numbers. The gateway confirms in $M_{3}$ that the sensor node is the real AID, because that message proves that the sensor node knows b0, b1, and KP(idx). It also confirms that the sensor node has the correct $r_{1}$ and $r_{2}$. Similarly, the sensor node confirms in $M_{4}$ the identity of the gateway and the accuracy of the random numbers. As can be seen, the principals prove their identities and validate the data trustworthiness before using it to update AID, preventing this type of attack.

#### 5.3.17. Resistance to Key Disclosure Attack

*Resistance to long-term key disclosure attack*: The long-term key of the sensor node $k_{s}$, of the gateway $k_{g}$, and the shared key between them KP(idx), are never sent in cleartext, ciphered with an xor, or transformed in another invertible way. Keys $k_{s}$ and $k_{g}$ are only used to construct b0 and b1, after hashing them with random numbers. Similarly, b0, b1, and KP(idx) are sent in the public channel only after hashing them with the secret random numbers $r_{1}$ and $r_{2}$. Therefore, because it is infeasible to invert the hash output, the adversary cannot obtain or construct $k_{s}$, $k_{g}$, and KP(idx).

*Resistance to the Session-Key Disclosure Attack*: The session key is never sent in a message. It is constructed by the principals using $r_{1}$, $r_{2}$, and KP(idx), which are unknown to the adversary. The random numbers $r_{1}$ and $r_{2}$ are sent in the public channel using the hashed b0 and b1 to cipher them. Thus, the adversary cannot obtain them. Additionally, the key KP(idx) is sent only after hashing it with $r_{1}$, $r_{2}$, timestamps, b0, and b1. Therefore, the adversary can neither obtain nor construct the session key.

## 6. Performance and Security Evaluation

In this section, a performance analysis of LAKD protocol is presented. Five authentication protocols with similar architectures to the proposed scheme were used for comparison. The followed methodology is similar to the work in [59], where the performance was based on the execution-time and communication-cost of the protocols. The next two subsections present the execution-time and communication-cost analysis, respectively.

### 6.1. Execution-Time Analysis

In the execution-time calculation, what is contemplated is the cost of executing hash functions and cipher algorithms; the cost of very lightweight operations, such as xor, addition, and subtraction is considered negligible. Two metrics were used for the hashing and encrypting/decrypting cost, similar to [59]. They are described in Table 4. The first metric, denominated Case 1, corresponds to the work in [21]. The second metric, named Case 2, is from [33].

The execution-cost of LAKD protocol is presented in Table 5, together with that of protocols [22,60,61,62,63], which were also designed for M2M communication. Concerning the Case 1 measurement, the proposal has less execution-time than the other protocols, except for the schemes of Esfahani et al. [22] and Joshitta et al. [63]. The cost difference between LAKD and Esfahani et al. is of just one more hash execution in the sensor node in LAKD, and for Joshitta et al. the difference is of 48.07% less cost in the medical device compared to the sensor node of LAKD, and 23.07% less in the authentication server compared with the gateway of LAKD. For Case 2, only Esfahani et al. has less execution-time, because of the additional hash execution in the sensor node of LAKD.

The proposed protocol was designed to achieve two goals: proper security and computational-cost for resource-constrained IIoT devices. As can be seen in Table 5, the proposal has a proper execution-cost when comparing it with other schemes. Some protocols have less cost; however, they do not achieve all the security properties that LAKD protocol does. Two of them even have serious security issues, such as key disclosure, as is described in the subsection Attack Resistance Comparison.

### 6.2. Communication-Cost Analysis

The communication-cost was analyzed according to the number of bits that have to transmit the principals to authenticate, as in [44,59,64,65]. To compute the communication-cost, the size of each transmitted datum in the protocol was obtained and added to get the total amount of transmitted bits. Two metrics were used to obtain the size of the transmitted data, similarly to the methodologies in [44,59]. They are described in Table 6. In the first metric, denominated Case 1, it is considered that hash outputs, random numbers, timestamps, identities, and keys are 128 bits long, and for Case 2 they are 256 bits long. In both cases, the encryption output is of 128 bits per block [59]. The sizes of 128 and 256 bits of the metrics follow the conventional size of data in security.

Table 7 presents the communication-cost of LAKD protocol and the comparison schemes. Three protocols have less communication-cost than the proposal, which are Esfahani et al., Han et al., and Joshitta et al., with differences of 33.33%, 41.67%, and 50% (54.17% for Case 2), respectively. The proposed protocol requires sending only four messages to achieve mutual authentication, and they contain timestamps, xor-ciphered random numbers, and message authentication codes (MACs) from a hash function. Sending this information prevents attacks such as modification, replay, DoS, and impersonation [28]. The protocols that have less communication-cost do not send all this information; consequently, they have been found vulnerable to some of the attacks, as can be seen in the subsection Attack Resistance Comparison. LAKD protocol has a low communication-cost when compared with Qiu et al. and Renuka et al., and a proper cost compared to Esfahani et al., Han et al., and Joshitta et al. Therefore, even if the proposal is not the protocol with the less communication-cost, it has a proper cost for IIoT, and it does achieve more security properties than the protocols with less cost. We developed our proposal giving security more priority than saving a few bits in the transmission.

### 6.3. Attack Resistance Comparison

The security properties of LAKD protocol and the other schemes for M2M communication were analyzed to obtain their attack resistance. Furthermore, articles that have a security analysis of the comparison schemes were reviewed to complete the information, such as [28,29,30]. In Table 8 is summarized the analysis results of common attacks, as was done in [44,45,64,65]. Through analyzing the attack resistance of an authentication protocol, researchers and developers can decide its best application area, and they can be aware of its limitations and how to handle them.

As can be seen in Table 8, schemes with less execution-time and communication-cost have security vulnerabilities that our proposal does not. Take Esfahani et al.’s protocol, for example. It has been found vulnerable to modification, session and long-term key disclosure, privileged insider, impersonation, DoS, and tracking attacks in [28,29,30]. Concerning Joshitta et al., some of its security vulnerabilities are the use of the same session key between sessions, and its disclosure.

## 7. Discussion

In this paper, an authentication protocol called LAKD is proposed for IIoT devices. It aims for low computing requirements to enable its implementation in highly resource-constrained devices, such as those of Class 0 in the RFC 7228 classification. To accomplish it, the protocol does not use any public-key cryptography, because using it has a high impact on the device’s resources [15,16]. Additionally, it does not use encryption algorithms; instead, it is based only on simple operations such as xor, addition, subtraction, and a hash function. According to the measurements in [21], the hash function has 75.93% less execution-time than an encryption algorithm, and in [33] it had 99.85% less.

LAKD protocol uses the xor operation to cipher session random numbers, and a hash function to generate MACs to have integrity of the transmitted data. The use of these lightweight operations makes the protocol have a low impact on the resources of the devices, and still achieves the security properties required for IIoT. As was described in section Security Analysis, the security of the protocol was formally and informally analyzed. One formal analysis was presented using the AVISPA tool, a well-known instrument that has been used in many protocols to assess their security. It considers a Dolev–Yao channel where the adversary can intercept, reassemble, and modify any message, which represents real adversary capabilities. In that scenario, LAKD protocol was concluded to be safe against replay and MITM attacks, which are the verifications the tool performs. Another formal analysis was done using BAN logic. The authentication was complete when the next goals were accomplished:

$$GW|\equiv (GW\overset{SK}{⟷}SN).$$

$$SN|\equiv (GW\overset{SK}{⟷}SN).$$

And a strong authentication was achieved when the following goals were met [66]:

$$GW|\equiv SN|\equiv (GW\overset{SK}{⟷}SN).$$

$$SN|\equiv GW|\equiv (GW\overset{SK}{⟷}SN).$$

An informal analysis was also presented, demonstrating the accomplishment of the security properties of confidentiality, integrity, mutual authentication, sensor node anonymity, perfect forward and backward secrecy, and known session key security, and its resistance against known attacks. Table 8 is an attack-resistance comparison between the proposal and protocols for M2M communication with similar architecture. It showed that LAKD protocol achieves security similar to schemes that require more computational resources, such as Qiu et al., which is resistant to many attacks but at the cost of a high execution-time and communication-cost.

Of the security properties achieved by the proposed protocol, sensor node anonymity and tracking resistance have special importance in IoT. If an adversary can know the node or user behind some activities, private information can be exposed, such as medical situations, work routines, living habits, etc. This information can be used from targeted marketing to extortion. The LAKD protocol accomplishes anonymity and tracking resistance through working with pseudonyms in the sensor node instead of its identification, and by changing the pseudonym in each session to prevent the adversary from associating activities to a specific node, similarly to Qiu et al. Additionally, the use of random numbers makes messages different and unpredictable in each session, which impedes the adversary in finding a relationship between them.

Security and performance in terms of execution-time and communication-cost were contrasted. In Table 9 are the differences in the percentages of execution-times of the schemes against LAKD, and in Table 10 of the communication-cost. As can be seen, secure proposals such as Qiu et al. have higher execution-times and communication-costs. Comparing the proposal to Qiu et al., the sensor node, which is the most constrained device, has 63% less execution-time for Case 1, and 99.69% for Case 2. Also, it transmits 71.43% fewer bits. These savings are significant for resource-constrained devices, where the limited resources have to be shared between the IoT application, and the network and security services.

As with every protocol, there are limits in LAKD. Its main limitation is that it is required that the gateway store a key pool for each sensor node in its network. Usually, the gateway is a device with plenty of computational resources. However, if its network is very large, the storage cost can be significant. An approach to managing this for large networks is having an authentication server that stores all the key pools. Then, when a sensor node starts the communication with the gateway, the latter retrieves from the authentication server the sensor node’s key pool to perform the authentication. The gateway could store some key pools of sensor nodes that frequently communicate with it, similarly to the cache memory proposed in [67], to prevent delays in their communication.

## 8. Conclusions

In this work, the LAKD authentication protocol for M2M communication in IIoT has been presented. It intends for a low computational-cost to be suitable for resource-constrained IIoT devices. To achieve it, the proposal is based on the lightweight operations xor, addition, and subtraction, and a hash function. The security of the protocol was assessed with the AVISPA tool and BAN logic, which confirmed its mutual authentication goal, and its resistance against replay and MITM attacks. Also, the accomplishments of confidentiality, integrity, mutual authentication, perfect forward and backward secrecy, and known session key security were informally analyzed. Additionally, its resistance to known attacks was checked. The performance of the proposal was analyzed and compared to schemes for M2M communication with similar architecture. It resulted in a good execution-time and communication-cost for IIoT. When comparing the security, LAKD resulted in higher attack resistance, its security being similar to schemes with more computational requirements.

The high security and low computational-cost of LAKD protocol allow resource-constrained IIoT devices to be capable of implementing a security service to protect data privacy and industrial secrets, and prevent threats such as device impersonation, data disclosure, and MITM attacks, which can disrupt the system operation, and even threaten the user’s welfare.

## Figures and Tables

**Table 1:** LAKD protocol notation.

Symbol	Description
$k_{g}$	Secret key of the gateway.
$ID_{g}$	Identity of the gateway.
$k_{s}$	Secret key of the sensor node.
$ID_{s}$	Identity of the sensor node.
AID	Pseudonym of the sensor node.
b0, b1	Secret values shared by the gateway and sensor node.
KP	Key pool of the sensor node.
idx	A key index of the key pool of sensor node.
ΔT	Predefined maximum acceptable delay for message reception.
*h*	One-way hash function.
⊕	Xor function.
||	Concatenation operator.

**Table 2:** Burrows–Abadi–Needham (BAN) logic notation.

Symbol	Description
P,Q	Principals.
X,Y	Statements.
*K*	Encryption key.
P∣≡X	*P* believes *X*.
P◃X	*P* sees *X*.
P∣∼X	*P* once said *X*.
P⇒X	*P* has jurisdiction over *X*.
♯(X)	*X* has not be sent in a previous protocol execution (it is fresh).
$P\overset{K}{⟷}Q$	*P* and *Q* may use key *K* in their communication.
$P\overset{X}{⇌}Q$	*X* is a secret which is known only by *P* and *Q*, and possibly by principals that they trust.
${\left\{X\right\}}_{K}$	*X* is encrypted using key *K*.
${\langle X\rangle }_{Y}$	*X* combined with *Y*, where *Y* is a secret which proves the identity of the one who sent it.

**Table 3:** BAN logic rules.

	Symbol	Description
(1)	$\frac{P|\equiv Q\overset{K}{⟷}P,P◃{\left\{X\right\}}_{K}}{P|\equiv Q|∼X}$	Message-meaning rule.
	$\frac{P|\equiv Q\overset{Y}{⇌}P,P◃{\langle X\rangle }_{Y}}{P|\equiv Q|∼X}$	
(2)	$\frac{P|\equiv ♯(X),P|\equiv Q|∼X}{P|\equiv Q|\equiv X}$	Nonce-verification rule.
(3)	$\frac{P|\equiv Q\Rightarrow X,P|\equiv Q|\equiv X}{P|\equiv X}$	Jurisdiction rule.
(4)	$\frac{P|\equiv ♯(X)}{P|\equiv ♯(X,Y)}$	If one part of a formula is fresh, then the entire formula is fresh.
	$\frac{P|\equiv X,P|\equiv Y}{P|\equiv (X,Y)}$	
(5)	$\frac{P|\equiv (X,Y)}{P|\equiv X}$	Belief rule.
	$\frac{P|\equiv Q|\equiv (X,Y)}{P|\equiv Q|\equiv X}$	

**Table 4:** Execution-time comparison criteria. The cost is presented in milliseconds (ms). $T_{h}$ represents a hash function execution, and $T_{e}$ the AES encryption/decryption execution.

	Function	Cost (ms)
Case 1	$T_{h}$	0.0051700
	$T_{e}$	0.0214800
Case 2	$T_{h}$	0.0000328
	$T_{e}$	0.0214385

**Table 5:** Execution-time comparison.

Protocol	Principal	Operations	Case 1	Case 2
Esfahani et al. [22]	Sensor node	$7T_{h}$	0.03619 ms	0.0002296 ms
	Router	$8T_{h}$	0.04136 ms	0.0002624 ms
Han et al. [60]	Device 1	$3T_{e}$	0.06444 ms	0.0643155 ms
	Device 2	$3T_{e}$	0.06444 ms	0.0643155 ms
Qiu et al. [61]	Host	$5T_{h}+4T_{e}$	0.11177 ms	0.0859180 ms
	Router	$T_{h}+2T_{e}$	0.04813 ms	0.0429098 ms
	Edge router	$6T_{h}+6T_{e}$	0.15990 ms	0.1288278 ms
Renuka et al. [62]	Sensor C	$4T_{e}$	0.08592 ms	0.0857540 ms
	Sensor D	$3T_{e}$	0.06444 ms	0.0643155 ms
	Gateway	$3T_{e}$	0.06444 ms	0.0643155 ms
Joshitta et al. [63]	Medical device	$T_{e}$	0.02148 ms	0.0214385 ms
	Authentication server	$2T_{h}+T_{e}$	0.03182 ms	0.0215041 ms
LAKD	Sensor node	$8T_{h}$	0.04136 ms	0.0002624 ms
	Gateway	$8T_{h}$	0.04136 ms	0.0002624 ms

**Table 6:** Communication-cost comparison criteria.

	Data Size in Bits
Case 1	128
Case 2	256

**Table 7:** Communication-cost of the protocols in bits.

Protocol	Case 1	Case 2
Esfahani et al.	1024	2048
Han et al.	896	1792
Qiu et al.	5376	10752
Renuka et al.	3584	7168
Joshitta et al.	768	1408
LAKD	1536	3072

**Table 8:** Comparison of the protocols’ resistances to attacks.

Attack	Esfahani et al.	Han et al.	Qiu et al.	Renuka et al.	Joshitta et al.	LAKD
Tracking	✗	✗	✓	✗	✗	✓
Off-line identity guessing	✗	✗	✓	✗	✗	✓
Impersonation	✗	✓	✓	✓	✗	✓
MITM	✓	✓	✓	✓	✗	✓
Privileged insider	✗	✓	✓	✗	✗	✓
Replay	✓	✓	✓	✓	✗	✓
Known session-specific	✗	✓	✓	✓	✗	✓
temporary information						
DoS	✗	✗	✗	✗	✗	✓
Modification	✗	✗	✓	✓	✗	✓
Key disclosure	✗	✓	✓	✓	✗	✓

✓: The protocol is resistant to the attack. ✗: The protocol is vulnerable to the attack.

**Table 9:** The differences in percentages of execution-times of the schemes against LAKD.

Protocol	Principal	Case 1	Case 2
Esfahani et al.	Sensor node	12.50%	12.50%
	Router	0%	0%
Han et al.	Device 1	−35.82%	−99.59%
	Device 2	−35.82%	−99.59%
Qiu et al.	Host	−63.00%	−99.69%
	Router	−16.37%	−99.39%
	Edge router	−74.13%	−99.80%
Renuka et al.	Sensor C	−51.86%	−99.69%
	Sensor D	−35.82%	−99.59%
	Gateway	−35.82%	−99.59%
Joshitta et al.	Medical device	48.07%	−98.78%
	Authentication server	23.07%	−98.78%

**Table 10:** The differences in percentages of communication-cost for each scheme against LAKD.

Protocol	Case 1	Case 2
Esfahani et al.	33.33%	33.33%
Han et al.	41.67%	41.67%
Qiu et al.	−71.43%	−71.43%
Renuka et al.	−57.14%	−57.14%
Joshitta et al.	50%	54.17%

## Appendix A. BAN Logic Proof

From Message 1 of the idealized form of LAKD protocol, we obtain:

Step 1: $GW◃{\left\{r_{1},T_{1}\right\}}_{b1},{\left\{{\langle GW\overset{r_{1}}{⇌}SN\rangle }_{b0},T1\right\}}_{b0}$.
Step 2: From Step 1, applying Rule 1 and Assumptions 4 and 5, we get: $GW|\equiv SN|∼(r_{1},T_{1},{\langle GW\overset{r_{1}}{⇌}SN\rangle }_{b0},T_{1})$.
Step 3: From Step 2, applying Rule 4 and Assumption 2, we get: $GW|\equiv ♯(r_{1},T_{1})$.
Step 4: From Step 3, applying Rule 2, we get: $GW|\equiv SN|\equiv (r_{1},T_{1})$.
Step 5: From Step 4, applying Rule 3 and 5 and Assumption 1, we get: $GW|\equiv r_{1}$.
Step 6: From Step 2, applying Rule 4 and Assumption 2, we get: $GW|\equiv ♯({\langle GW\overset{r_{1}}{⇌}SN\rangle }_{b0},T_{1})$.
Step 7: From Step 6, applying Rule 2, we get: $GW|\equiv SN|\equiv (GW\overset{r_{1}}{⇌}SN,T_{1})$.
Step 8: From Step 7, applying Rule 3 and 5 and Assumption 1, we get: $GW|\equiv GW\overset{r_{1}}{⇌}SN$.

From Message 2, we obtain:

Step 9: $SN◃{\left\{r_{2},T_{2}\right\}}_{r_{1}},{\left\{{\langle GW\overset{r_{2}}{⇌}SN\rangle }_{r_{1}}\right\}}_{b1},{\left\{{\langle GW\overset{KP(idx)}{⇌}SN\rangle }_{r_{1}},r_{2}\right\}}_{b0}$.
Step 10: From Step 9, applying Rule 1 and Assumptions 9 and 10, we get: $SN|\equiv GW|∼(r_{2},T_{2},{\langle GW\overset{r_{2}}{⇌}SN\rangle }_{r_{1}},{\langle GW\overset{KP(idx)}{⇌}SN\rangle }_{r_{1}},r_{2})$.
Step 11: From Step 10, applying Rule 4 and Assumption 7, we get: $SN|\equiv ♯(r_{2},T_{2})$.
Step 12: From Step 11, applying Rule 2, we get: $SN|\equiv GW|\equiv (r_{2},T_{2})$.
Step 13: From Step 12, applying Rule 3 and 5 and Assumption 6, we get: $SN|\equiv r_{2}$.
Step 14: From Step 10, applying Rule 4 and the assumption SN $|\equiv ♯r_{1}$ because it is its originator, we get: $SN|\equiv ♯{\langle GW\overset{r_{2}}{⇌}SN\rangle }_{r_{1}}$.
Step 15: From Step 14, applying Rule 2, we get: $SN|\equiv GW|\equiv GW\overset{r_{2}}{⇌}SN$.
Step 16: From Step 15, applying Rule 3 and Assumption 6, we get: $SN|\equiv GW\overset{r_{2}}{⇌}SN$.
Step 17: From Step 10, applying Rule 4 and the assumption SN $|\equiv ♯r_{1}$, we get: $SN|\equiv ♯({\langle GW\overset{KP(idx)}{⇌}SN\rangle }_{r_{1}},r_{2})$.
Step 18: From Step 17, applying Rule 2, we get: $SN|\equiv GW|\equiv {\langle GW\overset{KP(idx)}{⇌}SN\rangle }_{r_{1}}$.

From Message 3, we obtain:

Step 19: $GW◃{\left\{GW\overset{SK}{⟷}SN,T_{3}\right\}}_{b1}$.
Step 20: From Step 19, applying Rule 1 and Assumption 5, we get: $GW|\equiv SN|∼(GW\overset{SK}{⟷}SN,T_{3})$.
Step 21: From Step 20, applying Rule 4 and Assumption 2, we get: $GW|\equiv ♯(GW\overset{SK}{⟷}SN,T_{3})$.
Step 22: From Step 21, applying Rule 2, we get: $GW|\equiv SN|\equiv (GW\overset{SK}{⟷}SN,T_{3})$.
Step 23: From Step 22, breaking the conjunction, we get: $GW|\equiv SN|\equiv GW\overset{SK}{⟷}SN$. **(Goal 3)**
Step 24: From Step 23, applying Rule 3, Assumptions 1 and 3, and the deductions of steps 5 and 8, we get: $GW|\equiv GW\overset{SK}{⟷}SN$. **(Goal 1)**

From Message 4, we obtain:

Step 25: $SN◃{\left\{GW\overset{SK}{⟷}SN,T_{4}\right\}}_{b0}$.
Step 26: From Step 25, applying Rule 1 and Assumption 9, we get: $SN|\equiv GW|∼(GW\overset{SK}{⟷}SN,T_{4})$.
Step 27: From Step 26, applying Rule 4 and Assumption 7, we get: $SN|\equiv ♯(GW\overset{SK}{⟷}SN,T_{4})$.
Step 28: From Step 27, applying Rule 2, we get: $SN|\equiv GW|\equiv (GW\overset{SK}{⟷}SN,T_{4})$.
Step 29: From Step 28, breaking the conjunction, we get: $SN|\equiv GW|\equiv GW\overset{SK}{⟷}SN$. **(Goal 4)**
Step 30: From Step 29, applying Rule 3, Assumptions 6 and 8, and the deductions of steps 13 and 16, we get: $SN|\equiv GW\overset{SK}{⟷}SN$. **(Goal 2)**

The four security goals that LAKD protocol has to satisfy to have mutual authentication are achieved.
